# Brace modifications that can result in improved curve correction in idiopathic scoliosis

**DOI:** 10.1186/1748-7161-9-2

**Published:** 2014-03-05

**Authors:** Grant Wood

**Affiliations:** 1 Align Clinic, LLC, 700 South Claremont Street, Suite 105, San Mateo, CA 94402, USA

**Keywords:** Scoliosis brace failures, TLSO brace, Cheneau-Rigo brace, Pad placements

## Abstract

**Background and aim:**

The purpose of this paper is to share with scoliosis professionals the X-rays of different pad placement levels associated with improved curve correction in a case of idiopathic scoliosis (IS). Scoliosis braces of all types and brands utilize common principles of construction that ensure good fit and function. Equally important to the end result is good patient follow-up care and brace quality control by the orthotist.

**Design and methods:**

This report reviewed the case of an 11-year-old girl diagnosed with IS, focusing on the in and out-of-brace x-rays, as well as the fit and function of the braces. The first brace was a TLSO-type, the second a Cheneau-type brace using a B1 model following the Rigo classification of scoliosis.

**Results:**

The first TLSO-type brace presented an in-brace X-ray that showed a curve increase. The Cheneau-type scoliosis brace reduced the Cobb angles over 50%.

**Conclusions:**

The biomechanical changes consequent to modifications in brace design and pad placements appeared to have improved the scoliosis and reduced the Cobb angles in this case. An orthotist must provide optimal fit and function of the brace which was prescribed by the referring physician. Adherence to certain basic design principles, and close follow up by the orthotist-especially during growth spurts - are critical to its effectiveness. Specifically, a skilled orthotist must be experienced with the particular brace-type, apply these principles, maintain a good working relationship with both physician and patient to ensure timely brace adjustments essential to continued brace comfort and efficacy.

## Background

The purpose of this paper is to share with scoliosis professionals the X-rays of different pad placement levels associated with improved curve correction in a case of idiopathic scoliosis (IS). Scoliosis braces of all types and brands utilize common principles of construction that ensure good fit and function. Equally important to the end result is good patient follow-up care and brace quality control by the orthotist.

## Case presentation

An 11-year-old girl diagnosed with idiopathic scoliosis with right thoracic and left lumbar Cobb angle curves of 22 degrees each. She was originally prescribed a thoracolumbosacral orthosis (TLSO) type brace and later prescribed a Cheneau-Rigo handmade type scoliosis brace. The Cheneau-Rigo handmade type brace [1] was a B1 type model using the Rigo classification of scoliosis [2].

It is the responsibility of the orthotist to provide an optimal fitting and functioning scoliosis brace. These qualities can be checked by the orthotist in many ways, according to rigorous standards set either by the individual orthotist with many years of experience in the conservative treatment of scoliosis as listed below or, for some practitioners, in accordance with standards established by the 2011 SOSORT guidelines [3].

• Visual check of the brace quality (i.e. the correct design for specific curve pattern).

• Palpating the spine inside the brace (possible in some brace types) to feel that the spine is straighter and pressures are at the correct levels.

• In-brace X-rays, as prescribed by the doctor (check that the amount of correction for the specific scoliosis is acceptable).

• In-brace clinical presentation (how the patient looks in the brace). The patient should look better clinically in the brace.

• Out-of-brace clinical presentation: Compare the current out-of-brace clinical presentation with pre-brace clinical presentation. Check for sagittal normalization, reduced rotation as well as pelvis and trunk alignments (body alignment).

## Method

The differences between the original Cheneau brace and the author’s Cheneau-Rigo handmade type brace are the following:

1. The brace was designed using the Rigo Classification of scoliosis and brace design.

2. The new Cheneau brace follows the current design shapes taught by Manuel Rigo, MD. Thus, it is a Cheneau-Rigo handmade type brace.

3. The brace was handmade by the author and it is the author’s personal version of the Cheneau-Rigo brace, thus the name follows the evolution of the brace, Wood Cheneau Rigo (WCR) brace.

Good fit and function today does not guarantee a good fitting and functioning brace in 6 or 12 months. This has to be considered and checked by close control and follow-up with the patient.

A scoliosis brace has many pads, pressures, reliefs, expansions, opening in many planes and orientations, and it is beyond the scope of this paper to discuss the optimal situation for each. Rather, it is to discuss how brace design and pad placements were changed in this particular case to improve curve correction of IS.

A finished TLSO scoliosis brace, of all names and brands, should be designed and finished with some basic standards which are imperative to having a more successful result for the patient for the next 9 to 12 months, and not only for the initial in-brace X-ray. For example, the thoracic pad that is one or two vertebra below the apex may produce a good initial in-brace correction but could cause progression later on if not monitored closely.

Thus, for most cases it is preferable to have the thoracic trimline (posterior right side of brace for a right curve) at the apex of the thoracic curve or, in the case of single curves, above the apex of the curve. This must be carefully fit by the orthotist to allow maximum pressures below the apex of the thoracic curve (but not too low), while leaving the actual brace or superior thoracic trimline above the center of thoracic pressure. Optimum thoracic curve correction is achieved when the maximum pressure is below the apex of the thoracic curve. The ribs below the apex actually push the curve above that point, therefore a brace pad placed below the apex pushes on the ribs that push and correct the curve at the apex [4]. However, the fixation of having the pad below the apex should be considered only if realistically possible. For example, this is not always possible if the thoracic apex is low and or in short thoracic curves with high lumbar curves. It must be considered that a pad 2 vertebra below the apex could cause the pad to block correction of that curve and of the lumbar curve. The 2 vertebra below the apex in this situation would have a pad that not only pushes the thoracic curve but would also push against the lumbar correction. The soft tissue below the apex will compress and the forces are not always totally transferred to the vertebra connected to those ribs, but rather to the spine at the level of the pressure. As a result, the low thoracic pad can block correction of the thoracic and lumbar curves if not placed correctly.

Scoliosis braces are fit to growing children who experience significant growth spurts that can make the brace too short relatively soon after that fitting. Therefore, the patient’s potential growth is considered when deciding on the level of pad placement.

Poor trunk decompensation was also observed in some patients, and although it does not directly cause curve progression, it presents the patient with a poor clinical presentation that should be addressed. However this issue is beyond to scope of this paper.

The typical course of bracing intervention for scoliosis is 2-4 years (depending on maturity of each patient), often spanning the child’s growth spurt. Several braces are typically needed to accommodate this growth and also to take advantage of the opportunity to augment the corrective forces after an initial break-in phase. The number and spacing of these braces depend also on scoliosis correction, patient acceptance and economic factors. The important point here is that whenever it is determined that the patient will remain in a single brace for a year or so, especially during the growth-spurt years, care must be taken to ensure that the thoracic pad does not end up applying forces too low on the spine toward the end of that bracing period.

**Figure 1:**
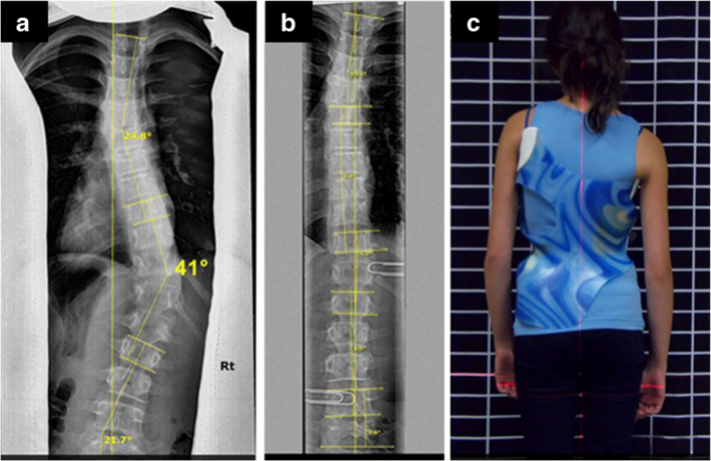
**WCR in-brace correction from 41 degrees to 5 degrees Cobb angle. a:** The X-ray presents a right thoracic curve of 41 degrees Cobb angle which was measured from T9 to L2 with the curve apex at T11-12. The upper thoracic curve was approximately 25 degrees Cobb angle. **b:** An in-brace X-ray of patient with Cheneau-Rigo handmade type brace, 18 hours after initial fitting. The thoracic trimline was as high as T10 (above the curve apex) and the pressure pad pushed from T10 to L3, which was above and below the measured Cobb angle. However, the center of maximum pressure was at T-12, as marked by the paperclip in the X-ray. A support force or slight counter force was placed on the left, at the level of L-4 and a left axilla force was applied at the maximum inclined vertebra at approximately T6. This was on the high side, however it provides room for the patient to grow and still provide optimal correction. Care must be taken in these cases to not produce a structural upper thoracic curve. The right thoracic curve reduced in-brace from 41 degrees to 5 degrees Cobb angle and the upper thoracic curve reduced in-brace from approximately 25 degrees Cobb angle to 10 degrees Cobb angle. **c:** Patient in a Cheneau-Rigo handmade type brace with optimal axilla, thoracic and lumbar pad heights.

**Figure 2:**
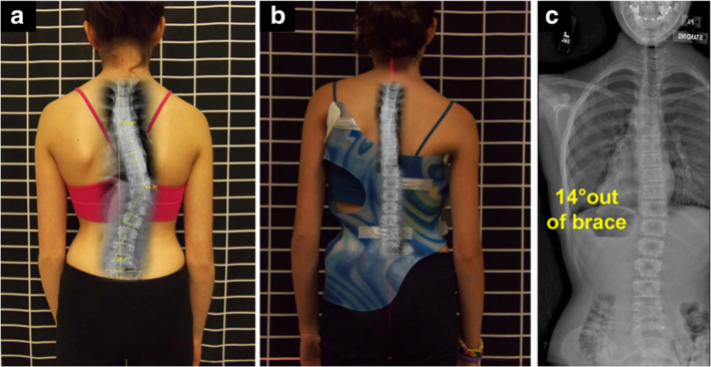
**WCR out-of-brace correction from 41 degrees to 14 degrees Cobb angle. a:** The clinical presentation of the patient with a right thoracic curve of 41 degrees Cobb angle. **b:** In-brace X-ray of patient with Cheneau type brace 18 hours after initial fitting. The right thoracic curve reduced in-brace from 41 degrees to 5 degrees Cobb angle and the upper thoracic curve reduced in-brace from approximately 25 degrees Cobb angle to 10 degrees Cobb angle. **c:** Out-of-brace X-ray showed a reduction in the major curve from 41 degrees prebrace to 14 degrees out-of-brace.

**Figure 3:**
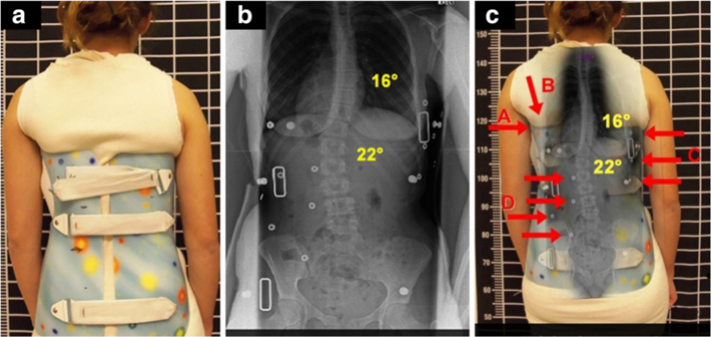
**TLSO-type brace with low axilla, thoracic and lumbar pads. a:** Patient (Nov 2013) in a TLSO-type brace which the patient reported to be a more or less comfortably fitting brace. **b** and **c:** In-brace X-ray of patient in TLSO type brace at one month presented right thoracic curve with the apex at T8 and left lumbar curve with the apex at L2. The thoracic pad was located at T10 to L1 and the lumbar pad was at L1 to L5 which could be considered theoretically correct. However in practical terms, these were too low when considering that only 2 cm to 3 cm of growth would cause these curves to go into progression. The in-brace X-ray presents a reduction of the curves from pre-brace of 22 degrees Cobb to in-brace 16 degrees Cobb and lumbar pre-brace of 27 degrees Cobb to in- brace of 22 degrees Cobb. The author fabricated a Cheneau-Rigo handmade type brace which had in brace correction from pre-brace thoracic 22 degrees Cobb to thoracic 3 degrees Cobb and pre-brace lumbar 27 degrees Cobb to 19 degrees in-brace Cobb.

**Figure 4:**
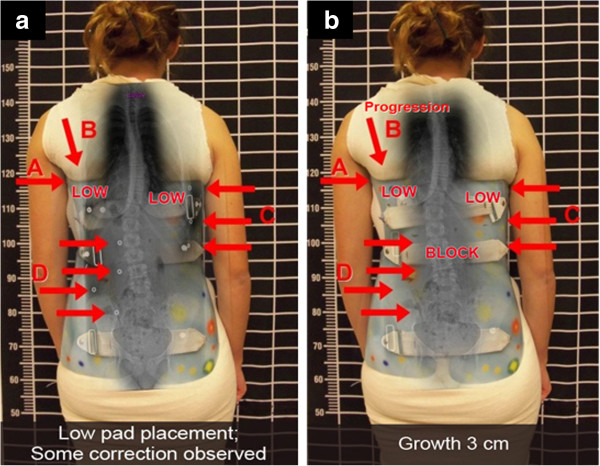
**a)TLSO-type brace with low pads that cause poor correction, b)a hypothetical situation of 3cm growth of the spine, therefore the brace now causes curve progression. a:** The patient after one month in her new TLSO type brace with common pad placements levels. **b:** This is a hypothetical situation that was created by altering the figure to demonstrate what could occur if the patient had a 3 cm growth of the spine (which could happen in a few weeks). The thoracic (C) and axilla (A) pads are low, which causes the scoliosis to go into progression (B), resulting in a poor outcome. The progression is caused by the counterforce of the left axilla extension (A) being too low and therefore directed towards the concave center of the curve, producing a buckling effect (B). The low thoracic pad (C) now blocks the lumbar correction (D).

Figures 1 and 2 show the correct thoracic and axilla forces required for thoracic Cobb angle correction in the coronal plane. If the brace is fit with a low thoracic trimline, it could produce negative effects subsequent to a significant and normal growth spurt (Figures 3 and 4).

**Figure 5:**
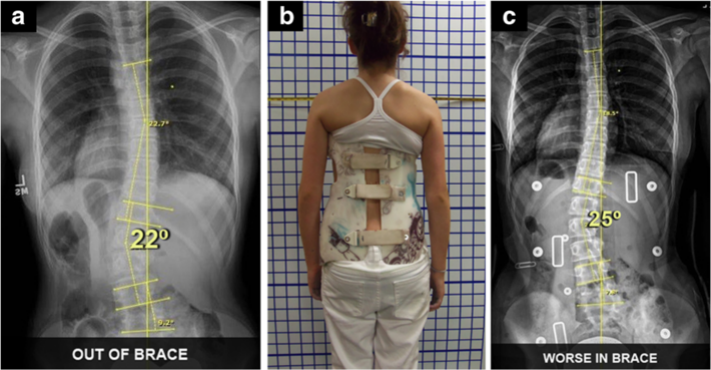
**TLSO-type brace with low axilla, thoracic and lumbar pads which presents a worse in-brace situation. a:** Patient out-of-brace on July 2013 presented with a right thoracic curve of 22-degree Cobb angle at T7 to T12 with the curve apex at T9; and a left lumbar curve of 22-degree Cobb angle at T12 to L4 with the curve apex at L2-3. Although the upper end-plate maximum inclination was T7, the curve inclined up to T7-T6-T5. **b:** Patient on July 2013 in TLSO-type brace which the patient reported to be a comfortably fitting brace. **c:** The TLSO-type in-brace X-ray presented the thoracic pad at T10 with the pad pressures and size of pad covering approximately T10 to T12/L1, and the counterforce at the left axilla at T 7-8. As a result, the in-brace X-ray showed a scoliosis which was worse in-brace compared to out-of-brace. TLSO-type in-brace was thoracic 18.5 degree Cobb and lumbar 25 degree Cobb angles. This was the direct result of firstly, the axilla extension being too low, which caused the buckling effect, and secondly, the low thoracic pad blocking the lumbar correction. This configuration was causing lumbar curve progression in the brace.

**Figure 6:**
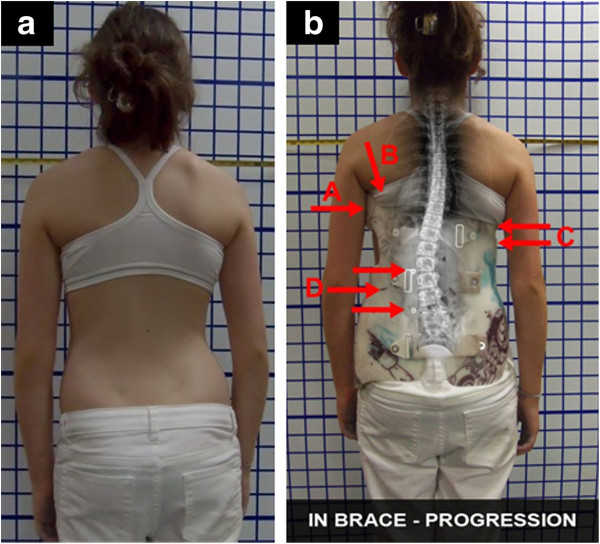
**TLSO-type brace with low axilla, thoracic and lumbar pads which causes scoliosis progression. a:** The clinical presentation of the patient with a right thoracic curve of 22-degree Cobb angle and a left lumbar curve of 22-degree Cobb angle. **b:** The in-brace X-ray showed a scoliosis which was worse in-brace compared to out-of-brace; TLSO-type in-brace was thoracic 22-Cobb and lumbar 25-degree Cobb angles. This was the direct result of firstly, the axilla extension being too low (A), which caused the buckling effect (B), and secondly, the low thoracic pad (C) blocking the lumbar correction (D). Furthermore, it was causing lumbar curve progression in the brace.

Pad placements that could cause scoliosis curve progression are presented in Figures 5a to 6b:

Some common causes of curve progression are listed below.

1. Thoracic pad and trimlines are left too low below the curve apex at the initial fitting of the scoliosis brace. This often presents with good in-brace correction. However, lack of follow up and/or brace replacement during the growth spurt eventually leaves a brace that is much too short for the patient, and thus causes progression, or at least less than optimal in-brace correction.

2. Axilla extension is low and therefore once the patient grows, it does not provide an optimal 3-point pressure system. This, in turn, may lead to a buckling or collapse into the concave side of the curve.

3. The thoracic pad is too low and blocks lumbar correction.

A scoliosis brace should be replaced prior to it actually being too short for the patient (i.e. at 11 to 12 months of treatment; in some cases before that). A short-fitting brace will most likely cause curve progression.

Some ways to provide an optimally-functioning brace that would last at least 9 months to 12 months, in most cases, are the following:

1. For standard TLSO type braces, the thoracic trimline is left to be at the level of the apex, but the pad is placed below the thoracic apex. This way, the brace is positioned such that as the patient grows, the thoracic pad can be placed higher.

2. In cases where there might not be good patient follow-up, the pad could be placed at the apex of the thoracic curve, thereby establishing optimal correction after approximately 3 or 4 months of brace wear (after the patient grows taller).

3. In other TLSOs that do not have pads, the thoracic pressure is applied from the brace itself. In these cases it would be desirable to leave the trimline high at the apex (some curve patterns should go above the apex). For optimal Cobb angle correction, the brace should be flared out at the apex and slightly below it, keeping the main pressures below the apex of the thoracic curve.

**Figure 7:**
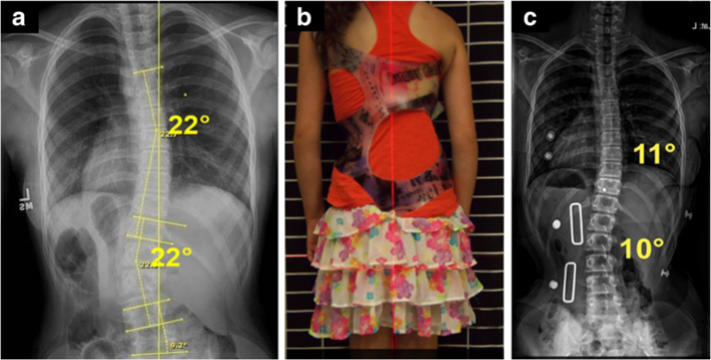
**WCR in-brace correction with optimal pad placements. a:** Patient out-of-brace and pre- Cheneau-Rigo handmade type brace in July 2013, showing a right thoracic curve of 22-degree Cobb angle at T7 to T12 with the curve apex at T9; and a left lumbar curve of 22-degree Cobb angle at T12 to L4 with the curve apex at L2-3. Although the upper end-plate maximum inclination was T7, the curve inclined up to T7-T6-T5. **b:** Patient in a Cheneau-Rigo handmade type brace, with correction of decompensation of the trunk and a more balanced pelvis. **c:** The Cheneau-Rigo handmade type in-brace X-ray presented the thoracic pad at T9-10 with the pad pressures and size of pad covering approximately T8 to T11 and the counterforce at the left axilla at T 5. As a result, the Cheneau-Rigo handmade type in-brace X-ray showed a scoliosis with over 50% correction, which improved from thoracic 22 degrees Cobb and lumbar 22 degrees Cobb out-of-brace to thoracic 11 degrees Cobb and lumbar 10 degrees Cobb in-brace.

**Figure 8:**
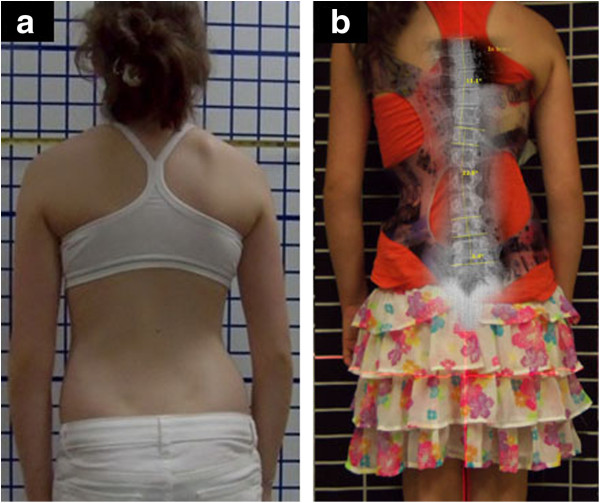
**WCR brace with improved clinical presentation of patient. a:** The clinical presentation of the patient with a right thoracic curve of 22-degree Cobb angle and a left lumbar curve of 22-degree Cobb angle. **b:** X-ray of patient in the Cheneau-Rigo handmade type brace showed over 50% correction of scoliosis, which improved from thoracic 22 degrees Cobb and lumbar 22 degrees Cobb out-of-brace to thoracic 11 degrees Cobb and lumbar 10 degrees Cobb in-brace.

Independent of the brace type, the levels of vertebral pressures have to be correct to allow 3-point pressure systems to effect optimal lateral translation of each section of the spine. These pressures must be designed to open the concave side of the lateral curves as demonstrated in Figures 7 and 8.

## Results

The Cheneau-Rigo handmade type scoliosis brace reduced the respective thoracic and lumbar Cobb angles from 22 degrees and 22 degrees to 11 degrees and 10 degrees.

## Conclusion

The Biomechanical changes consequent to these changes in brace design and pad placements appeared to have improved the scoliosis and reduced the Cobb angles.

An orthotist must provide optimal fit and function of the brace which was prescribed by the referring physician. The function of the scoliosis brace will affect the patient not only during the course of treatment, but also for the patient’s entire life. Therefore, effective conservative treatment of scoliosis requires that the brace meet basic standards essential for good fit and function and that the orthotist maintain close patient follow up care and brace quality control, especially during the patient’s growth spurts. The orthotist must be experienced in the particular brace type prescribed by the MD and be diligent in the follow up care, thus ensuring that timely and appropriate brace quality-control adjustments are made.

### Consent

Written informed consent was obtained from the parents of patients for publication of this Case Report and any accompanying images. A copy of the written consent is available for review by the Editor-in-Chief of this journal.

## Abbreviations

IS: Idiopathic scoliosis; TLSO: Thoracolumbosacral orthosis; WCR: Wood Cheneau Rigo.

## Competing interests

The author declares that he has no competing interests.

## Authors’ contributions

GW conceived the study, handmade the Cheneau-Rigo scoliosis braces, drafted the manuscript and gave final approval of the version to be published.

## Authors’ information

GW is the Co-founder and CEO of Align Clinic in San Mateo, CA. Author of the Wood Cheneau Rigo (WCR) scoliosis brace. The Current Chair of the Spinal Orthotics Society of the American Academy of Orthotists and Prosthetist. Member of SOSORT. American board certified in Orthotics. UK Certified in prosthetics and orthotics. Master of Science by thesis on the Cheneau brace.

Grant Wood: http://www.align-clinic.com
